# Hyperlactataemia and Hypocalcaemia as Predictors of Mortality in Adult Polytrauma Patients: A Prospective Observational Study

**DOI:** 10.7759/cureus.117447

**Published:** 2026-10-04

**Authors:** Reeti Sharma, Zia Arshad, Vinod Srivastav, Ravi Prakash, Yadvendra Dheer

**Affiliations:** 1 Anesthesiology and Critical Care, King George's Medical University, Lucknow, IND; 2 Trauma Surgery, King George's Medical University, Lucknow, IND

**Keywords:** apache ii, hyperlactataemia, hypocalcaemia, injury severity score, intensive care unit, mortality, polytrauma, serum calcium, serum lactate, sofa score

## Abstract

Background: Early identification of high-risk polytrauma patients remains a major challenge in critical care. This study aimed to evaluate the prognostic value of serum lactate and serum calcium in predicting in-hospital mortality among adult polytrauma patients admitted to the intensive care unit (ICU).

Methods: One hundred adult polytrauma patients aged 18-65 years with an Injury Severity Score (ISS) >16 and admitted within 48 hours of injury were enrolled. Baseline serum calcium and serial arterial lactate levels were measured during ICU stay. ISS, Acute Physiology and Chronic Health Evaluation II (APACHE II), Sequential Organ Failure Assessment (SOFA) score, days of mechanical ventilation, ICU stay, and mortality were noted.

Results: Overall in-hospital mortality was 62%. Non-survivors demonstrated significantly higher serial serum lactate concentrations throughout ICU admission than survivors (p < 0.001). Baseline serum lactate showed a significant negative correlation with survival (r = -0.379, p < 0.001), while peak lactate demonstrated an even stronger association (r = -0.498, p < 0.001). Baseline serum calcium was significantly associated with mortality and demonstrated inverse correlations with APACHE II, SOFA, and ISS, indicating that lower calcium levels were associated with greater injury severity and poorer clinical outcomes. Peak serum lactate also correlated positively with established severity scoring systems.

Conclusions: Hyperlactataemia and hypocalcaemia are significant predictors of mortality in critically ill polytrauma patients. Both biomarkers correlate closely with validated trauma severity scores and provide valuable prognostic information early during ICU admission.

## Introduction

Polytrauma represents a significant global health burden and is a major contributor to mortality and long-term disability, particularly among young and economically active individuals. Rapid urbanisation, increased motorisation, and industrial growth have resulted in a rising incidence of severe traumatic injuries, with road traffic accidents continuing to be the predominant mechanism of injury in many developing countries. Patients with multiple traumatic injuries frequently present with complex and rapidly evolving physiological disturbances, necessitating early recognition, timely resuscitation, and appropriate critical care. Although advances in prehospital services, organised trauma systems, surgical management, and intensive care have improved the overall care of severely injured patients, mortality remains considerable, especially during the initial period following trauma. Consequently, early identification of trauma patients at increased risk of poor outcomes remains a key priority for optimizing management and improving survival [1].

Accurate prognostication in polytrauma traditionally relies on validated scoring systems such as the Injury Severity Score (ISS), Acute Physiology and Chronic Health Evaluation II (APACHE II), and Sequential Organ Failure Assessment (SOFA) score. Although these tools are valuable for assessing injury severity and predicting clinical outcomes, they require multiple physiological and laboratory variables and may not always be readily available during the initial resuscitation period. Consequently, simple biochemical biomarkers that can rapidly identify critically ill patients have gained increasing attention as adjuncts to conventional trauma scoring systems [2].

Serum lactate is one of the most widely investigated biomarkers in critically ill patients. Elevated lactate levels primarily reflect tissue hypoperfusion, impaired oxygen delivery, and increased anaerobic metabolism following severe injury. Persistent hyperlactataemia has been associated with ongoing shock, inadequate tissue perfusion, multiple organ dysfunction, prolonged intensive care stay, and increased mortality. In addition to its role as a marker of metabolic stress, serial lactate measurements and lactate clearance have been proposed as useful indicators of response to resuscitation, enabling clinicians to monitor therapeutic effectiveness during the early stages of trauma management [3].

Calcium is another critical physiological determinant in trauma patients. It plays an essential role in myocardial contractility, vascular smooth muscle function, neuromuscular transmission, and activation of the coagulation cascade. Hypocalcaemia frequently develops following severe trauma because of blood loss, impaired calcium homeostasis, and citrate-containing blood product transfusion. Reduced serum calcium concentrations can aggravate trauma-induced coagulopathy, impair cardiovascular performance, and contribute to persistent hypotension and tissue hypoperfusion. Recent evidence has identified hypocalcaemia as an important component of the "lethal diamond" of trauma resuscitation, emphasizing its prognostic significance alongside hypothermia, acidosis, and coagulopathy [4].

Although both hyperlactataemia and hypocalcaemia have independently been associated with poor outcomes after trauma, limited prospective evidence has evaluated their combined prognostic value in adult polytrauma patients admitted to the intensive care unit. Understanding the relationship between these readily available biochemical markers and established severity scores may facilitate earlier identification of patients at increased risk of mortality, allowing timely escalation of monitoring, resuscitation, and organ support. Such biomarkers may also complement existing prognostic models by providing rapid and objective information during the initial management of critically ill trauma patients [5,6].

Accordingly, this prospective observational study was conducted to assess the association between baseline and peak serum lactate levels, as well as baseline serum calcium concentration, and in-hospital mortality among adult polytrauma patients admitted to the intensive care unit. The study also evaluated the relationship of these biochemical markers with established trauma severity scores, duration of mechanical ventilation, and ICU length of stay. By examining these readily measurable biomarkers, the study aims to generate clinically relevant evidence regarding the potential role of serum lactate and calcium as simple, cost-effective, and accessible prognostic indicators for early risk stratification and optimisation of management in patients with severe polytrauma.

## Materials and methods

Study design and setting

This prospective observational study was conducted in a tertiary care hospital and teaching centre over a period of 12 months (August 2025-August 2026). The study evaluated the prognostic significance of serum lactate and serum calcium levels in adult polytrauma patients admitted to the intensive care unit (ICU). The study protocol was approved by the Institutional Ethics Committee, and all procedures were performed in accordance with the ethical principles of the Declaration of Helsinki (2013) and the guidelines of the Indian Council of Medical Research (ICMR). The Clinical Trials Registry-India (CTRI) registration number was CTRI/2025/10/096472. Written informed consent was obtained from the legally authorised representative of each patient before enrolment.

Adult patients aged 18-65 years presenting with polytrauma and admitted to the ICU within 48 hours of injury were screened for eligibility. Polytrauma was defined as an ISS > 16. Consecutive eligible patients were recruited using non-probability consecutive sampling until the required sample size of 100 patients was achieved. The sample size was calculated using the formula for estimation of a single population proportion and was increased to 100 participants to account for potential loss to follow-up.

Patients aged 18-65 years with a history of polytrauma and ICU admission within 48 hours of injury were included in the study. Written informed consent was obtained from a legally authorised family member. Patients were excluded if they died within 48 hours of ICU admission or were pregnant. Individuals with conditions known to influence serum lactate or calcium levels, including diabetes mellitus, coronary artery disease, chronic renal failure, liver dysfunction, alcoholism, malignancy (particularly lymphoma), epilepsy, seizures, acute pancreatitis, or those receiving medications such as epinephrine, metformin, or nucleoside analogues for human immunodeficiency virus (HIV) infection, were also excluded. Patients who left against medical advice before completion of the study were excluded from the final analysis.

Eligible patients were prospectively followed throughout their ICU stay, from admission until discharge or death. Demographic characteristics, mechanism of injury, haemodynamic status on admission, and clinical parameters were recorded using a standardised data collection proforma. Disease severity was assessed at ICU admission using the ISS, APACHE II, and SOFA scores [7-9]. Arterial blood samples were collected immediately after ICU admission to measure serum lactate concentration using point-of-care blood gas analysis. Subsequent arterial lactate measurements were performed every 24 hours during the ICU stay to evaluate serial lactate trends and determine the peak serum lactate concentration. Venous blood samples obtained at admission were analysed for baseline serum ionised calcium concentration using standard laboratory methods available in the hospital biochemistry laboratory.

The primary outcome of the study was in-hospital mortality. Secondary outcomes included the correlation of baseline serum calcium and serum lactate concentrations with established severity scores, including ISS, APACHE II, and SOFA, as well as the duration of mechanical ventilation and length of ICU stay. Serial serum lactate values were also analysed to evaluate their relationship with patient survival and disease severity during ICU admission.

Statistical analysis

Data were entered into a computerised database and analysed using IBM SPSS Statistics for Windows, Version 28.0 (Released 2021; IBM Corp., Armonk, NY, USA). Continuous variables were summarised as mean ± standard deviation (SD), while categorical variables were expressed as frequencies and percentages. The distribution of continuous variables was assessed prior to statistical analysis. For comparison between groups, the independent-samples t-test was used for normally distributed continuous variables, whereas the Mann-Whitney U test was applied for non-normally distributed variables. Categorical variables were compared using Fisher’s exact test. Associations between serum lactate, serum calcium, trauma severity scores, and clinical outcomes were evaluated using Pearson’s or Spearman’s correlation coefficients, as appropriate based on the distribution of the data. All statistical tests were two-sided, and a p-value < 0.05 was considered indicative of statistical significance.

## Results

Baseline characteristics

A total of 100 adult polytrauma patients admitted to the ICU were included in the study. The mean age of the study population was 37.28 ± 13.6 years, with the majority of patients (63%) belonging to the 18-40-year age group, while the remaining 37% were between 41 and 65 years of age. Male patients predominated, accounting for 83% of the study population, whereas females constituted 17% (Table 1).

**Table 1:** Demographic profile of the patients (N = 100)

	Number of patients, n (%)
Age group	
18-40	63 (63.0%)
41-65	37 (37.0%)
Mean ± SD	37.28±13.6
Gender	
Male	83 (83.0%)
Female	17 (17.0%)
Mechanism of trauma	
Road traffic accidents	97 (97.0%)
Physical assault	3 (3.0%)

Road traffic accidents were the principal mechanism of injury, accounting for 97% of all cases, while physical assault was responsible for only 3% of injuries. On admission, 45% of patients were haemodynamically stable, 54% required norepinephrine support because of haemodynamic instability, and 1% required both norepinephrine and vasopressin support. 

The study population represented patients with severe trauma. The mean ISS was 27.68 ± 4.6, indicating substantial anatomical injury. The mean APACHE II score was 19.3 ± 5.2, while the mean SOFA score at ICU admission was 4.95 ± 1.9, reflecting moderate physiological derangement and organ dysfunction (Table 2).

**Table 2:** Descriptive statistics (mean and standard deviation) of severity scoring systems among the studied patients ISS: Injury Severity Score, APACHE II: Acute Physiology and Chronic Health Evaluation II, SOFA: Sequential Organ Failure Assessment.

Variable	Mean	SD
ISS	27.68	4.6
APACHE II score	19.3	5.2
SOFA score	4.95	1.9

Clinical outcomes

Fifty-eight patients (58%) required mechanical ventilation for ≤7 days, whereas 42 (42%) patients required ventilator support for >7 days. The mean duration of mechanical ventilation was 7.20 ± 3.7 days.

Regarding ICU stay, more than half of the patients required prolonged critical care, reflecting the severity of injury and associated physiological disturbances. The overall in-hospital mortality rate was 62% (n = 62), while 38% (n = 38) of patients survived to hospital discharge (Table 3).

**Table 3:** Clinical parameters of the patients (N = 100) ISS: Injury Severity Score, APACHE II: Acute Physiology and Chronic Health Evaluation II, SOFA: Sequential Organ Failure Assessment.

	Number of patients, n (%)
Haemodynamic status on admission	
Stable	45 (45.0%)
Unstable on norepinephrine support	54 (54.0%)
Unstable on norepinephrine and vasopressin support	1 (1.0%)
Mechanical ventilation	
≤7 days	58 (58.0%)
>7 days	42 (42.0%)
Mean ± SD	7.20 ± 3.7
ICU stay	
≤7 days	55 (55.0%)
>7 days	45 (45.0%)
Mean ± SD	7.91 ± 3.8
Mortality	
Yes	62 (62.0%)
No	38 (38.0%)
Injury scores, mean (SD)	
ISS	27.68 (4.6)
APACHE II	19.3 (5.2)
SOFA	4.95 (1.9)

Serial serum lactate levels

Serial serum lactate measurements demonstrated consistently higher lactate concentrations among non-survivors compared with survivors throughout ICU admission. The differences between the two groups were statistically significant on nearly all observation days (p < 0.001), indicating persistent tissue hypoperfusion and metabolic stress among patients who ultimately died.

Baseline serum lactate showed a significant inverse correlation with survival (r = -0.379, p < 0.001). Furthermore, peak serum lactate demonstrated an even stronger negative correlation with survival (r = -0.498, p < 0.001), suggesting that increasing lactate levels were associated with progressively poorer clinical outcomes (Table 4).

**Table 4:** Comparison between lactate, calcium, and mortality **Correlation is significant at the 0.01 level (two-tailed Student's t test).

	Mortality	
	p-value	r-value
Baseline serum lactate	<0.001	-0.379^**^
			Peak serum lactate	<0.001	-0.498^**^
						Baseline serum calcium	<0.001	-0.563^**^

Baseline serum calcium

Baseline serum calcium concentration also demonstrated significant prognostic value. Lower calcium levels were associated with increased mortality, supporting the role of hypocalcaemia as an indicator of poor outcome in severe polytrauma.

Correlation analysis further showed significant inverse relationships between baseline serum calcium concentration and established severity scores. Serum calcium correlated negatively with APACHE II (r = -0.577, p < 0.001), SOFA (r = -0.295, p = 0.003), and ISS (r = -0.307, p =0.002). These findings indicate that patients with lower serum calcium concentrations generally presented with more severe physiological and anatomical injury.

Correlation between biomarkers and severity scores

Serum lactate and serum calcium showed distinct but complementary relationships with disease severity. Peak serum lactate demonstrated positive correlations with all major severity scoring systems, indicating that patients with greater injury burden and physiological dysfunction exhibited higher lactate concentrations (Tables 5-7).

**Table 5:** Comparison of serial serum calcium levels between survivors and non-survivors during ICU stay

Serum calcium	Mortality				p-value
	Yes (n = 62)		No (n = 38)		
	N	Mean ± SD	N	Mean ± SD	
Day 1	62	3.46 ± 0.51	38	4.11 ± 0.38	<0.001
Day 2	62	3.47 ± 0.48	38	4.12 ± 0.37	<0.001
Day 3	62	3.51 ± 0.52	38	4.07 ± 0.38	<0.001
Day 4	55	3.51 ± 0.47	34	3.97 ± 0.40	<0.001
Day 5	49	3.44 ± 0.74	29	4.06 ± 0.40	<0.001
Day 6	39	3.50 ± 0.35	26	4.08 ± 0.22	<0.001
Day 7	33	3.51 ±0.42	24	4.10 ± 0.31	<0.001
Day 8	27	3.49 ± 0.51	19	3.49 ± 0.36	0.002
Day 9	21	3.43 ± 0.36	17	4.08 ± 0.25	<0.001
Day 10	17	3.60 ± 0.32	15	4.08 ± 0.33	<0.001
Day 11	12	3.57 ± 0.37	15	3.99 ± 0.34	0.007
Day 12	9	3.65 ± 0.62	13	4.04 ± 0.46	0.115
Day 13	6	3.57 ± 0.36	8	4.02 ± 0.42	0.061
Day 14	4	3.49 ± 0.45	8	3.94 ± 0.35	0.089
Day 15	1	2.61 ± 0.01	7	3.97 ± 0.44	0.029
Day 16	1	2.80 ± 0.01	5	4.09 ± 0.38	0.037

**Table 6:** Comparison of serial serum lactate levels between survivors and non-survivors during ICU stay

Serum lactate	Mortality				p-value
	Yes (n = 62)		No (n = 38)		
	N	Mean ± SD	N	Mean ± SD	
Day 1	62	2.91 ± 1.0	38	2.0 ± 0.91	<0.001
Day 2	62	2.94 ± 1.2	38	1.84 ± 0.80	<0.001
Day 3	62	3.0 ± 1.2	38	1.74 ± 0.68	<0.001
Day 4	55	3.35 ± 1.5	34	1.74 ± 0.69	<0.001
Day 5	49	3.31 ± 2.3	29	1.63 ± 0.58	<0.001
Day 6	39	3.23 ± 1.6	26	1.62 ± 0.69	<0.001
Day 7	33	2.91 ± 0.70	24	1.58 ± 0.57	<0.001
Day 8	27	3.07 ± 0.70	19	1.62 ± 0.55	<0.001
Day 9	21	2.91 ± 0.79	17	1.58 ± 0.68	<0.001
Day 10	17	3.04 ± 0.70	15	1.63 ± 0.63	<0.001
Day 11	12	3.42 ± 0.75	15	1.44 ± 0.66	<0.001
Day 12	9	3.10 ± 1.1	13	1.42 ± 0.54	<0.001
Day 13	6	2.71 ± 1.2	8	1.64 ± 0.54	<0.001
Day 14	4	2.50 ± 1.0	8	1.03 ± 0.41	0.035
Day 15	1	2.19 ± 0.1	7	1.36 ± 0.39	0.099
Day 16	1	11.38 ± 0.1	5	1.31 ± 0.39	<0.001

**Table 7:** Correlation of APACHE II, SOFA, and ISS with serum calcium and lactate **Correlation is significant at the 0.01 level (two-tailed). ISS: Injury Severity Score, APACHE II: Acute Physiology and Chronic Health Evaluation II, SOFA: Sequential Organ Failure Assessment.

	APACHE II		SOFA		ISS	
	p-value	r-value	p-value	r-value	p-value	r-value
Serum calcium	<0.001	-.577^**^	0.003	-.295^**^	0.002	-.307^**^
Baseline serum lactate	<0.001	.364^**^	0.09	0.17	0.678	0.042
							Peak serum lactate	0.003	0.296	<0.001	0.378	<0.001	0.374

Conversely, baseline serum calcium showed negative correlations with these scoring systems, suggesting that worsening injury severity was accompanied by progressively lower calcium concentrations.

These findings support the hypothesis that hyperlactataemia reflects persistent tissue hypoperfusion and metabolic dysfunction, whereas hypocalcaemia reflects impairment of cardiovascular function and coagulation associated with severe trauma.

## Discussion

The present prospective observational study evaluated the prognostic significance of serum lactate and serum calcium in adult polytrauma patients admitted to the ICU. The findings demonstrate that both hyperlactataemia and hypocalcaemia are significantly associated with in-hospital mortality and correlate with established severity scoring systems, including the ISS, APACHE II, and SOFA scores. These observations support the utility of these readily available biochemical markers for early risk stratification in critically ill trauma patients.

The demographic profile of the study population was consistent with the epidemiology of major trauma reported in previous studies. The mean age of 37.28 years and the predominance of male patients reflect the greater exposure of young adult males to high-energy trauma, particularly road traffic accidents. In the present study, road traffic accidents accounted for 97% of injuries, emphasizing the continuing burden of traffic-related trauma in India. These findings are comparable with previously published trauma studies included in the literature review of this thesis, which similarly reported young males involved in road traffic accidents as the predominant patient population [10].

Serum lactate emerged as one of the strongest predictors of mortality in the present study. Both baseline and peak lactate levels demonstrated significant associations with survival, with peak lactate exhibiting a stronger correlation than admission values. Furthermore, non-survivors maintained significantly higher serial lactate concentrations throughout their ICU stay compared with survivors. These findings suggest that persistent hyperlactataemia reflects ongoing tissue hypoperfusion and inadequate metabolic recovery despite intensive care management [11,12].

The observed relationship between elevated lactate levels and adverse outcomes is consistent with previous studies. Several investigators have demonstrated that elevated admission lactate and delayed lactate clearance are associated with increased mortality, multiple organ dysfunction, prolonged intensive care stay, and greater transfusion requirements in trauma patients. The present findings further support the concept that serial lactate measurement provides greater prognostic information than a single admission value because it reflects the dynamic response to resuscitation and restoration of tissue perfusion. Persistent elevation of lactate may therefore identify patients requiring more aggressive haemodynamic optimization and organ support [13,14].

An important observation of this study was the significant association between baseline serum calcium concentration and mortality. Lower serum calcium levels correlated with higher APACHE II, SOFA, and ISS scores, indicating that hypocalcaemia was associated with increasing injury severity and physiological derangement. These findings are biologically plausible because calcium plays an essential role in myocardial contractility, vascular tone, coagulation, and intracellular signalling. Following severe trauma, hypocalcaemia may result from haemorrhage, impaired calcium homeostasis, and citrate administration during blood transfusion, thereby contributing to cardiovascular instability and trauma-induced coagulopathy [15].

The relationship observed between hypocalcaemia and mortality is in agreement with several studies summarized in the literature review, which have reported increased mortality among trauma patients with reduced serum calcium concentrations. Recent systematic reviews cited in the thesis have similarly concluded that hypocalcaemia is associated with increased mortality and greater transfusion requirements following major trauma. Although some investigators have questioned whether hypocalcaemia remains an independent predictor after adjustment for injury severity, the present study demonstrates a significant correlation between baseline calcium concentration and validated severity scores, supporting its usefulness as an early prognostic marker in critically ill polytrauma patients [16].

Another important finding was the complementary prognostic value of serum lactate and serum calcium. While elevated lactate reflects impaired tissue oxygen delivery and metabolic stress, reduced calcium concentration indicates haemostatic dysfunction and cardiovascular compromise. Together, these biomarkers provide information on different aspects of the pathophysiological response to trauma. Their combined assessment may therefore improve early identification of patients at high risk of deterioration beyond what can be achieved using conventional physiological scoring systems alone.

The correlations observed between these biomarkers and APACHE II, SOFA, and ISS further strengthen their clinical relevance. These established scoring systems are widely accepted predictors of mortality in critically ill patients but require multiple clinical and laboratory variables for calculation. In contrast, serum lactate and calcium measurements are inexpensive, rapidly available, and routinely performed in most emergency departments and intensive care units. Their integration into the initial assessment of polytrauma patients may therefore facilitate earlier clinical decision-making, improve triage, and support timely resuscitation strategies.

The high mortality rate observed in this study reflects the inclusion of patients with severe polytrauma requiring intensive care admission and substantial physiological compromise. The mean ISS and APACHE II scores indicate that the study population represented a critically ill cohort with a high burden of injury, which may explain the observed mortality. These findings reinforce the importance of early prognostic markers capable of identifying patients who are likely to require prolonged organ support and aggressive critical care interventions [17].

The present study has certain limitations. It was conducted at a single tertiary care centre and included a relatively modest sample of 100 patients, which may limit the generalizability of the findings. Serum calcium was measured only at baseline, whereas serial calcium measurements might have provided additional prognostic information. In addition, the observational study design precludes establishment of causal relationships between biochemical abnormalities and mortality. Future multicentre studies with larger patient populations and serial assessment of both biomarkers are warranted to validate these findings and to determine whether biomarker-guided resuscitation strategies can improve clinical outcomes.

Despite these limitations, the study has several strengths. The prospective design minimized recall bias, standardized clinical assessment using validated severity scores, and incorporated serial lactate measurements throughout ICU admission. The simultaneous evaluation of serum lactate and serum calcium provides clinically relevant evidence supporting their combined use as simple, readily available biomarkers for early prognostication in adult polytrauma patients.

Overall, the findings of the present study indicate that hyperlactataemia and hypocalcaemia are important indicators of injury severity and adverse clinical outcomes. Their routine assessment at ICU admission, together with serial lactate monitoring, may enhance early risk stratification, optimize resuscitation strategies, and contribute to improved management of critically ill patients with severe polytrauma.

## Conclusions

This prospective observational study demonstrated that both hyperlactataemia and hypocalcaemia are significant predictors of mortality in adult polytrauma patients admitted to the intensive care unit. Elevated baseline and peak serum lactate levels were strongly associated with poor clinical outcomes, while lower baseline serum calcium concentrations correlated with increased injury severity, organ dysfunction, and mortality. Furthermore, both biomarkers showed significant relationships with established prognostic scoring systems, including ISS, APACHE II, and SOFA.

The findings suggest that serum lactate and serum calcium are inexpensive, rapidly available, and clinically useful biomarkers that can complement conventional trauma severity scores during the early assessment of critically ill patients. Incorporating these laboratory parameters into routine trauma evaluation may facilitate earlier identification of high-risk patients, improve risk stratification, and support timely resuscitation and intensive care interventions. Larger multicentre studies are warranted to validate these findings and to determine whether biomarker-guided therapeutic strategies can improve survival in patients with severe polytrauma.
